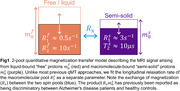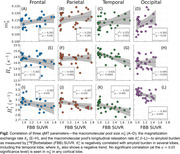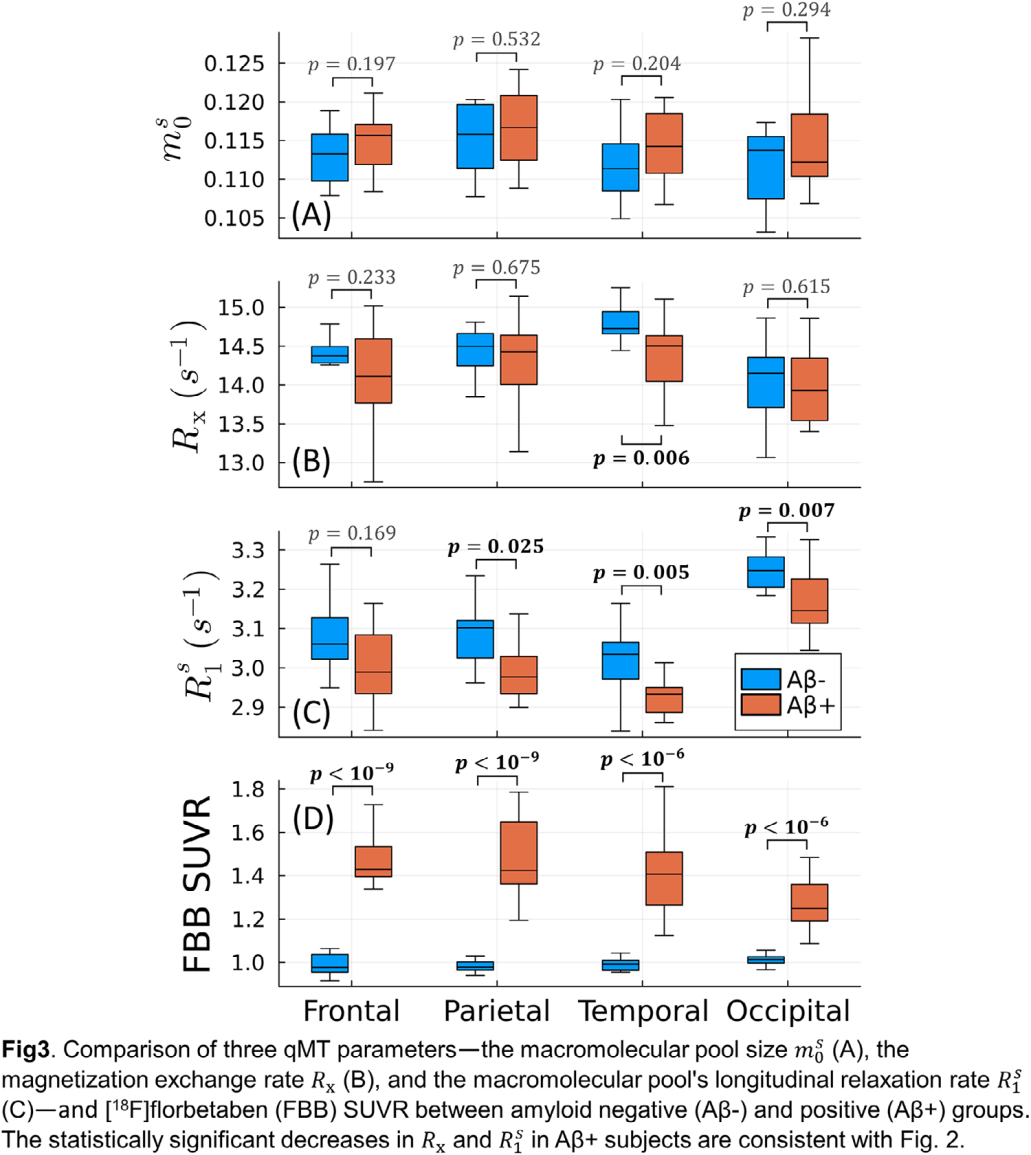# Feasibility of detecting Amyloid burden in preclinical Alzheimer’s disease with quantitative magnetization transfer MRI

**DOI:** 10.1002/alz.087669

**Published:** 2025-01-09

**Authors:** Andrew Mao, Sebastian Flassbeck, Elisa Marchetto, Henry Rusinek, Jakob Assländer

**Affiliations:** ^1^ NYU Grossman School of Medicine, New York, NY USA; ^2^ NYU Alzheimer's Disease Research Center, New York, NY USA

## Abstract

**Background:**

Magnetization transfer (MT) MRI is sensitive to the presence of macromolecules, including amyloid‐beta, and previous work suggests that it may be useful for discriminating patients with Alzheimer's disease (AD) from healthy controls. In this study, we investigated if quantitative MT (qMT) is capable of detecting the amyloid concentration in a preclinical cohort.

**Method:**

We recruited 14 subjects with a clinical dementia rating of 0 from NYU's ADRC cohort (7 male, mean age 74, 6 amyloid‐negative). An experimental 12 min qMT technique with an effective 1.24 mm isotropic resolution was used to obtain 6 parameter maps describing an unconstrained 2‐pool qMT model (Figure 1). MPRAGE and FLAIR images were acquired at 1 mm isotropic, rigid‐body registered to the qMT maps and used to perform Freesurfer‐based cortical parcellation. [^18^F]Florbetaben (FBB) images acquired within the preceding 24 months were rigid‐body registered to the qMT maps and used to compute average measurements across each cortical lobe for each hemisphere at 50% of the cortical thickness. Pearson's correlation coefficients were computed for all 6 qMT parameters with respect to FBB SUVR values. Amyloid‐positive and negative groups were compared using one‐way ANOVA tests. 5 Desikan‐Killiany ROIs containing substantial artifacts in the qMT maps were excluded from all analyses, though some imaging artifacts remain.

**Result:**

The exchange rate between the two spin pools and the macromolecular pool's longitudinal relaxation rate were found to be both negatively correlated with amyloid concentration and significantly decreased in amyloid‐positive subjects most prominently within the temporal lobe (Figures 2–3). The former is consistent with previous reports in more advanced AD stages, but the latter has not previously been reported as its value is fixed in most qMT methods. However, we observed no significant changes in the macromolecular pool size—which encompasses both myelin and amyloid‐beta—with increasing amyloid burden.

**Conclusion:**

MT MRI may be a promising tool for monitoring amyloid accumulation without the need for contrast agents or radiotracers. A larger cohort and a further optimized qMT sequence are needed to validate these preliminary findings.